# Staging of Cervical Lymph Nodes in Oral Squamous Cell Carcinoma: Adding Ultrasound in Clinically Lymph Node Negative Patients May Improve Diagnostic Work-Up

**DOI:** 10.1371/journal.pone.0090360

**Published:** 2014-03-20

**Authors:** Rikke Norling, Birgitte Marie Due Buron, Marianne Hamilton Therkildsen, Birthe Merete Henriksen, Christian von Buchwald, Michael Bachmann Nielsen

**Affiliations:** 1 Department of Radiology, Rigshospitalet, Copenhagen University Hospital, Copenhagen, Denmark; 2 Department of Pathology, Rigshospitalet, Copenhagen University Hospital, Copenhagen, Denmark; 3 Department of Otorhinolaryngology, Head & Neck Surgery and Audiology, Rigshospitalet, Copenhagen University Hospital, Copenhagen, Denmark; West Virginia University, United States of America

## Abstract

**Introduction:**

Clinical staging of patients with oral squamous cell carcinoma (OSCC) is crucial for the choice of treatment. Computed tomography (CT) and/or magnetic resonance imaging (MRI) are typically recommended and used for staging of the cervical lymph nodes (LNs). Although ultrasonography (US) is a non-expensive, accessible and non-ionising imaging modality this method is not consistently used.

This study aimed to investigate if addition of US of patients classified as clinically LN negative (cN0) by CT and/or MRI, increases the detection of LN metastases. Also, we aimed to identify which of the sonographic characteristics: echogenicity, border, shape, appearance of hilum and nodal blood-flow pattern best detect metastases in this patient group.

**Method:**

Fifty-one patients with OSCC classified as cN0 by CT/MRI were consecutively included and prospectively examined with US prior to sentinel node biopsy or selective neck dissection. Localisation, size and sonographic characteristics were registered for each LN and compared with the pathological findings. Sensitivity, specificity, positive predictive value (PPV) and negative predictive value (NPV) were calculated for different size measurements and sonographic characteristics.

**Results:**

We found that short axial diameter was the best size criterion for detection of metastases. However, the sonographic characteristics were better predictors than size and the presence at least four of the sonographic characteristics: hypo-echoic or heterogeneous appearance; irregular border; spherical shape; absence of nodal hilum; and peripheral nodal blood-flow resulted in a sensitivity of 43.8; specificity 91.4; PPV 70.0; and NPV 78.0. The number of patients with occult metastases decreased from 16 out of 51 (31%) to nine out of 51 (18%). Three patients (6%) were over-staged by US.

**Conclusion:**

The addition of US to the clinical work-up of patients with cN0 OSCC increases the detection of metastases, thus US potentially reduces the number of patients requiring a secondary neck surgery after sentinel node biopsy.

## Introduction

Oral squamous cell carcinomas (OSCC) constitute around 95% of oral cavity cancers [1] and predominantly spread to the lymph nodes of the neck. The presence of positive lymph nodes (LN) is a significant adverse prognostic factor for survival [2]. Between 10 and 52% of clinical LN negative (cN0) necks are pathological LN positive (pN+) [3]. Due to this high proportion of occult metastases, radical neck dissection or selective neck dissection are traditionally used for treatment of both cN0 and cN+ necks. However, the proportion of occult metastases depends on the sensitivity of the initial diagnostic methods employed e.g. palpation and imaging.

Computed tomography (CT), magnetic resonance imaging (MRI) and ultrasound (US) are widely recommended for the work-up of patients with head and neck cancer [4] and have shown comparable accuracies in patient populations consisting of both cN0 and cN+ patients [5]–[7]. CT is the most used modality followed by US in the Nordic countries [8]. In addition to the choice of imaging modality also the criteria for classifying the lymph node as cN+ are ambiguous. Size is the most widely used criterion but the sensitivity and specificity depend on the cut-off. For CT and MRI a 10–15 mm cut-off is commonly used. However, for US international consensus criteria to discriminate between cN0 and cN+ are absent [8]. Also, the optimal cut-off appear to depend on the whether it is a cN0 or cN+ patient population [9]. Along with size, other sonographic characteristics have been correlated with the presence of metastases: hypoechoic or heterogeneous internal structure, irregular LN border, absence of hilum [10], spherical shape and, peripheral nodal blood flow pattern [11].

In Denmark patients suspected of malignant tumour of the head and neck enter the national cancer pathways. The pathway for OSCC recommends CT and/or MRI of the primary tumour and the neck and may be supplemented with US of the neck [12]. However, US of the neck is not performed routinely at all centres [8]. In a treatment regimen with sentinel node biopsy the clinical staging is crucial since patients classified as cN0 undergo sentinel node biopsy (SNB) while patients with cN+ or advanced tumour-stages (T3–4) undergo selective neck dissection (SND). With the findings of metastasis at the histopathological examination of the SNB specimen the patients are offered a subsequent SND. The impact on prognosis of micrometastases defined as metastatic deposit less than or equal to 2 mm remains undetermined, since they cannot be detected by imaging [5], [13]. The primary aim of this study was to investigate if addition of US could increase the detection of LN metastases in patients classified as cN0 by CT and/or MRI. Secondly, we wanted to identify predictive sonographic characteristics in this selected patient group.

## Materials and Methods

### Patients

Fifty-one consecutive patients from the Department of Otorhinolaryngology, Head & Neck Surgery and Audiology, Copenhagen University Hospital, Rigshospitalet, Denmark were prospectively enrolled in the study in the period 1^st^ of April 2010 to 1^st^ of July 2012. The hospital serves as a tertiary Head & Neck centre for cancer treatment and receives patients from five referral hospitals serving a population of 2.5 million [14]. Patients with suspicion of or histopathologically verified OSCC were eligible. Patients with a history of radiation or surgical treatment to the head and neck were excluded. The patients followed the Danish national cancer pathways for head and neck cancer, which includes clinical examination including inspection, palpation, biopsy and nasal endoscopy and imaging. Subsequently patients participated at a multidisciplinary conference where final decision on the clinical staging and treatment was made. Only patients staged as cN0 and planned for surgical treatment of the neck were included.

The 51 patients (31 men, 20 women) had a mean age of 64.3 years (range 32–93). The primary tumour sites were floor of mouth in 26 patients (51%); anterior two-thirds of the tongue in 18 patients (35%); retromolar trigone in four patients (8%); and in the alveolar process in three patients (6%). Twenty-seven patients had a clinically (c)T1 tumour, 18 had cT2, two patients had cT3 and, four patients had cT4.

### Ethics statement

The study was approved by the local ethics committees (H-4-2010-011) and written informed consent was obtained from all patients.

### Ultrasonography

All patients were examined with US between 0 to 19 days before surgery (mean 4 days, median 1 day). The examination was performed by one trained observer (RN) under supervision of the chief radiologist (BMH), on a GE logiq 9 (GE Healthcare, Wisconsin, USA) using a GE 10L linear array transducer. Both sides of the neck were examined and all LNs measuring more than 3 mm [15] on the short axis were registered with regard to the following parameters: location [side and level]; size in three dimensions [length, long axial diameter and short axial diameter]; and five sonographic characteristics [echogenicity (hyper-, iso-, or hypo-echoic or heterogeneous appearance), surface border (regular, irregular), shape (oblong, oval or spherical), nodal hilum (present or absent) and nodal blood flow pattern detected with Power Doppler (not detectable, central, or peripheral flow pattern]. The location of the lymph nodes were divided into levels Ia, Ib, IIa, IIb, III, IV and V in accordance with the definition by Robbins et al. [16] and subsequently marked on an illustration of the neck.

Next, short axial diameter to length (S/L) ratio and ellipsoid volume were calculated for each LN. Ellipsoid volume was calculated as (4/3)*π*r_1_r_2_r_3_. US guided fine needle aspiration cytology (FNAC) was not a part of the study protocol.

### CT and MRI

Of the 51 patients, 40 patients underwent CT, 17 patients underwent MRI, and six patients had both CT and MRI. CT examinations were performed at the tertiary hospital or at the referral hospitals and all were performed with intravenous contrast and 3-mm axial images including coronal and sagittal multiplanar reconstructions. MRIs were performed at three referral hospitals and all included axial T1 and T2 weighted images and T1 weighted coronal and sagittal images of 3 to 6 mm intervals.

CT and MRI images were evaluated at a radiological conference prior to the multidisciplinary conference where final decision on the clinical staging and treatment was made. Patients who were staged as cN+ by imaging, defined as the presence of LNs larger than 15 mm on the axial short axis in level II and 10 mm in the remaining levels, were excluded from the analysis. For re-evaluation of the images two observers (RN and BB) blinded to the clinical information reviewed images concurrently on a picture archiving and communication system (PACS) (AGFA impax ES DS5300, Morstel, Belgium) reaching consensus on the radiological stage. Furthermore, for CT images LNs >3 mm on axial short axis were registered with regard to location, size in three dimensions, enhancement, and surface delineation. For MRI LNs >3 mm on the axial short axis were registered with regard to location, size in three dimensions, signal intensity including signs of central necrosis and surrounding oedema. The location of the LNs were registered in level Ia, Ib, IIa, IIb, III, IV and V in accordance with the “EORTC, GORTEC and RTOG endorsed consensus guidelines for the delineation of the CTV (clinical tumour volume) on the N0 neck of patients with head and neck squamous cell carcinoma” [17]. CT and MRI images were reviewed two months after inclusion of the last patient.

### Surgical treatment

At a multidisciplinary conference the patients were planned for surgical treatment with resection of the primary tumour and dissection of cervical LNs. The lymphadenectomies comprised SNB for patients with clinical early stage primary tumour (cT1 or cT2) and with no sign of neck metastases following palpation and CT and/or MRI examination (cN0). Patients with later stages cT3N0 or cT4N0 or with T-sites unsuitable for peritumoral injection underwent SND. SNB is considered less comprehensive than SND.

### Histopathology

All of the SNB specimens were examined in accordance with Sentinel European Node Trial, using step-serial sectioning at 150-micron intervals of the sentinel nodes [18]. Two sections from each level were stained with hematoxylin-eosin (H&E) and cytokeratin antibodies (AE1/AE3) and examined for tumour deposits with step serial sectioning of the sentinel nodes. The SND specimens were examined macroscopically and all palpable LNs were bisected and paraffin-embedded. Sections from each paraffin blocks were stained with H&E and the presence of metastases was determined by microscopic examination.

Comparison of the histopathology and US findings was done on a node-by-node basis pairing the side, level and size of the pathology-proven metastatic LN. In one case two metastatic LNs could not be paired with the US findings.

### Statistics

To investigate which size parameter is best suit for prediction of metastasis receiver operating characteristic (ROC) curves for different size parameters; short axial diameter, long axial diameter, length, short/length ration and ellipsoid volume (calculated as π/6×size in 3 dimensions) were generated. The sensitivity, specificity, positive predictive value (PPV) and negative predictive value (NPV) of different cut-off values of short axial diameter were calculated on a node-by-node basis, by coupling metastatic LN from the pathological findings with a LN at the same level identified with US. The coupling was made on basis of size and/or US characteristics.

To investigate the sonographic characteristics a logistic regression analysis was conducted to predict the presence of metastatic LNs using echogenicity, surface delineation, shape, nodal hilum and nodal blood flow pattern as predictors. For each of the five sonographic characteristics: echogenicity, surface delineation, shape, nodal hilum and nodal blood flow pattern the LN was registered as ‘suspicious’ if it appeared hypo-echoic or heterogeneous; had irregular border; spherical shape; absence of nodal hilum; and peripheral nodal blood flow.

In the evaluation of the effect of addition of US to CT/MRI in the clinical work-up, the sensitivity, specificity, PPV, NPV were calculated on a patient-by-patient basis. For each patient the LN with the most ‘suspicious’ characteristics was retrieve and correlated with the pathological status (pN0 or pN+)

## Results

**Figure 1:**
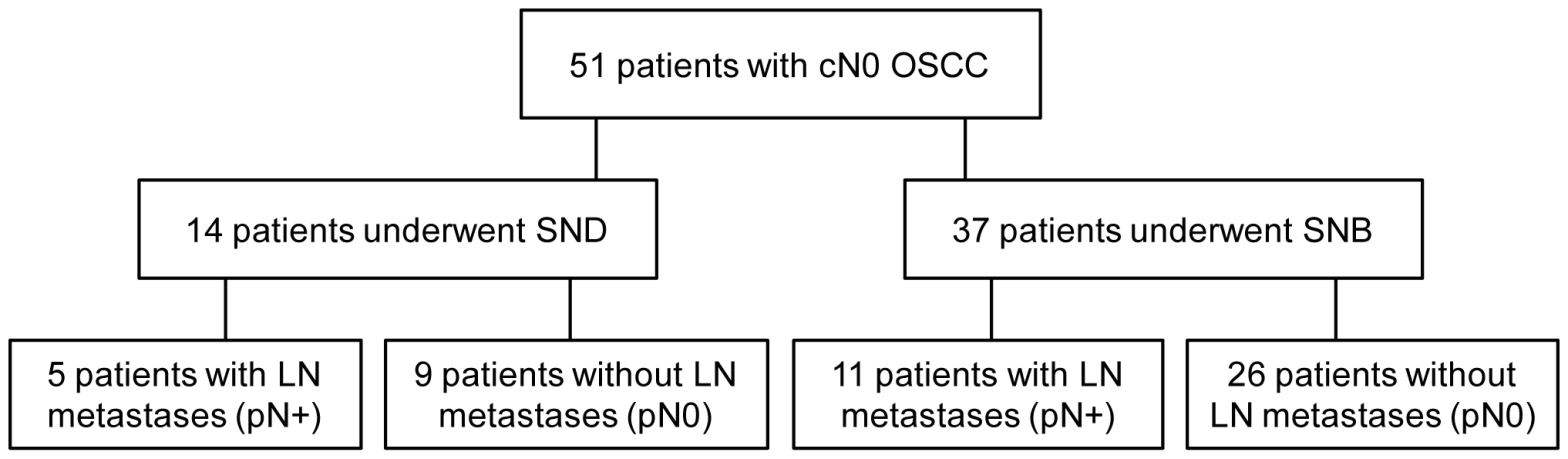
Flowchart for 51 patients who were clinically lymph node negative (cN0). Shows the distribution of neck treatment between selective neck dissection (SND) or sentinel node biopsy (SNB) and the subsequent pathological nodal findings: lymph node metastases (pN+) or pathologically lymph node negative (pN0).

Of the 51 patients, 14 patients underwent SND with a total of 296 retrieved LNs (range 4–84 per patient, mean 21, median 9). Histopathologically metastases were found in six LNs from five of these 14 patients (36%) (Figure 1). The remaining 37 patients underwent SNB procedures with retrieval of 109 dissected hot nodes (range 1–8 per patient, mean 3, median 3). Twenty-one of the 37 patients had -in addition to hot nodes- extra non-hot nodes removed during the procedure, counting 103 LNs (range 1–20 per patient, mean 5, median 4). Histopathologically metastases were found in 16 LNs (including five micro-metastases) in 11 (30%) of the 37 patients. Altogether 22 metastases (4%) were found among 508 retrieved LNs, consequential occult metastases (cN0→pN+) was seen in 16 (31%) of the 51 patients.

**Figure 2:**
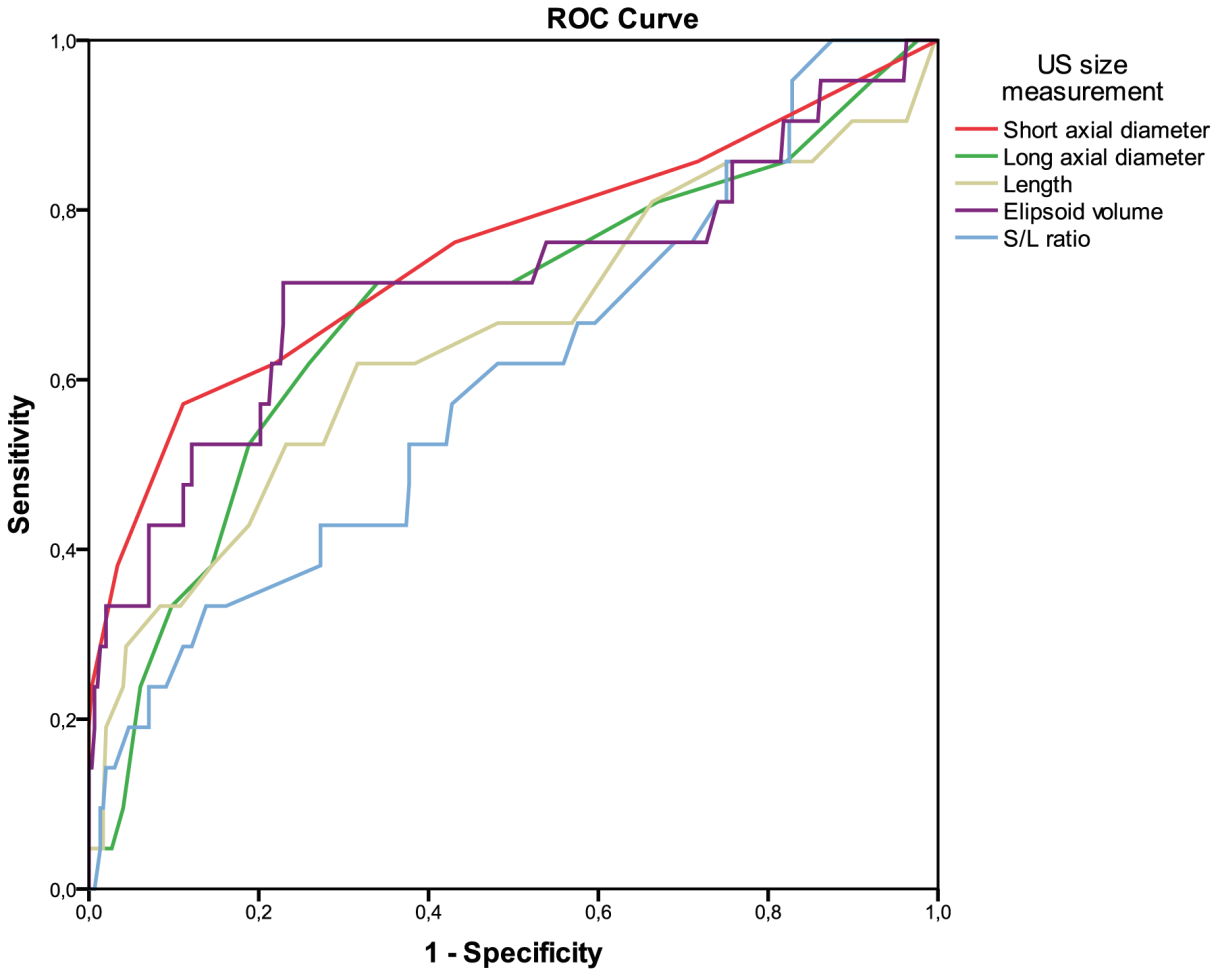
Receiver operating characteristic curves of ultrasonic size measurements. Shows receiver operating characteristic (ROC) curves for different ultrasonic size measurements: short axial diameter (area under curve, AUC: 0.752), long axial diameter (AUC. 0.685), length (AUC: 0.651), S/L ratio (AUC: 0.603) and ellipsoid volume (AUC: 0.717) as predictors of metastases on a node-by-node basis (N = 318).

To evaluate if addition of US to the clinical staging of OSCC increases the detection of LN metastases analysis were performed on a node-by-node basis for determination of cut-off for each of the sonographic characteristics. In total, 318 LN were identified by US among the 51 patients (median 6, range 2–19). First, ROC curves for different types of size criteria (short axis diameter, long axis diameter, length, short/length ratio and ellipsoid volume) were generated, showing that short axis diameter is the best size measurement for predicting metastases (Figure 2).

The short axial diameter has the largest area under the curve (0.752) followed by ellipsoid volume (0.717), long axial diameter (0.685), length (0.651) and S/L ratio (0.603).

To investigate if the cut-off for the short axial diameter is the same for all levels, the average of the size measurements were made on a level-by-level basis. The median short axial diameter for level IA, IB, IIA, IIB, III and IV was 4; 5; 5; 4; 4 and 4 mm respectively, showing that the cut-off for level IB and IIA should be 1 mm larger than for the rest of the neck. This difference in average size between levels was applied in the calculation of the sensitivity, specificity, PPV and NPV of different cut-off of short axial diameter (Table 1). It should be noted that the patient group was selected by having no LNs larger than 10 mm or 15 mm on CT/MRI.

**Table 1:** Sensitivity, specificity, PPV and NPV for different short axial diameter cut-offs.

Cut-off larger or equal to						
	4 mm	5 mm	6 mm	7 mm	8 mm	9 mm
Sensitivity	85.7	71.4	57.1	52.4	33.3	19.0
Specificity	38.0	64.3	83.8	92.9	99.0	100
PPV	8.9	12.4	20.0	34.4	70.0	100
NPV	97.4	97.0	96.5	96.5	95.5	94.6

Table 1 shows the sensitivity, specificity, PPV (positive predictive value) and NPV (negative predictive value) for different cut-off values of ultrasonically measured short axial diameter. A one millimetre larger cut-off was used for LNs in level IB and IIA.

As shown in Table 1 the sensitivity increases with decreasing cut-off but the PPV falls to a low level. Opposite if a cut-off of nine mm is chosen the PPV is 100%, but only finds 19% of the pN+. If the size criterion is applied on a patient-by-patient basis by retrieving the LN with the largest short axial diameter, a cut-off of 8 mm gives a sensitivity of 25.0, specificity 94.3, PPV 66.7, and NPV 73.3. This consequences that four of the 51 cN0 patients would be re-classified as cN+ while two patients would be over-staged as cN+.

As an alternative or supplement to the size-criterion we investigated which sonographic characteristics are the best predictors of LN metastasis in cN0 patients. A logistic regression analysis showed that hypo-echoic or heterogeneous appearance (odds ratio (OR): 1.2); irregular border (OR: 5.7); spherical shape (OR: 6.0); absence of nodal hilum (OR: 6.0); and peripheral nodal blood flow (OR: 5.2) are predictors of metastatic LNs.

To investigate if addition of US increases the detection of metastases the sensitivity and specificity was calculated on a patient-by-patient basis, by retrieving the LN with the most abnormal appearance from each patient and pairing this with the pathological status. With a cut-off representing the presence of at least four of the five above described characteristics US detected seven patients out of 51 (14%) with metastases who was initially classified as cN0 by CT/MRI. However, US also classified three (6%) patients as cN+ who was ultimately pN0. Sensitivity 43.8; specificity 91.4; PPV 70.0; and NPV 78.0. Therefore, with the addition of US subsequent to CT/MRI examination, the number of patients with occult metastases dropped from 16 out of 51 (31%) to nine out of 51 (18%).

## Discussion

The present study shows that adding US to the clinical work-up of cN0 OSCC increased detection of occult metastases in seven out of 51 patients (14%). This indicates that the implementation of US on a more routine base than currently recommended in guidelines may be valuable. Also, US resulted in a false positive finding in three out of 51 patients (6%). In a treatment regimen with SNB this would allocate the seven patients to SND and save the patients from a re-operation of the neck. For the three patients with false positive LN the consequence would be SND rather than SNB.

Due to the high incidence of occult metastases prophylactic neck dissection or radiotherapy is traditionally performed in patients who are cN0 to achieve regional control. The presence of occult metastases ranges between 10 to 52% [3]. This wide range may reflect that differences in diagnostic methods employed for clinical staging. In general, a risk of >10% or >20% for the presence of occult metastases is considered the threshold for performing prophylactic treatment of the neck [1], [19]. Current imaging methods do not assure a sufficiently high sensitivity, although US guided FNAC results in a sensitivity of around 90% in a mixed cN0 and cN+ population. However, using US guided FNAC in a cN0 population the sensitivity decreases to 73–78% [20], [21]. Furthermore, the sensitivity and specificity of the imaging depend on the radiological criteria for classifying the LN as cN+ and international consensus on these criteria are absent. The most commonly used criterion is LN size although cut-off varies [9]. In agreement with other studies we found that the short axial diameter is the best measurement of size to predict presence of metastases [9]. In contrast, we did not replicate that the S/L ratio could predict the presence of metastases. This may be explained by the inclusion in the present analysis of all visible LN in level 1 which are normally more rounded [10]. For US of cN0 patients, cut-offs of 7 mm for level 2 (sensitivity 77%, specificity 77%) and 6 mm for the remaining levels (sensitivity 81%, specificity 63%) have been proposed [9]. In the present study a similar cut-off resulted in a sensitivity of 52% and specificity of 93%. The discrepancies may be explained by differences in patient samples. In the present study inclusion was based on radiological criteria whereas van den Brekel et al [9] staged by palpation of the neck.

Micro-metastases are less than 2 mm and constitute a major challenge to radiological staging [22], [23]. In the present study pathology revealed four patients with micrometastases, and one patient with both macro- and micrometastasis. None of the micrometastases were detected by US. The visualisation of micro-metastases and small LNs is limited by the imaging resolution. Furthermore, the number of LN identified at diagnostic imaging is lower than the number of dissected LNs. For example, in colon cancer, the number of pericolonic LNs visualised by high-frequency in vitro US is less than half of what is found by colorectal resection and even less (16%) for LNs <5 mm [24], but could be increased with 3D-ultrasound [25]. In a previous study on cadavers we reported that 63% of normal cervical LNs are <3 mm [26]. Thus, in a cN0 population with no obvious enlargement of the LNs only a minor proportion can be expected to be visualized even with a high-frequency US on LNs of the neck. With the exclusion of micro-metastases the rate of occult metastases could be decreased to 10% (five out of 51) with the method described.

We intended to investigate clinically pragmatic sonographic characteristics and their application. Different LN characteristics have been described to correlate with the presence of metastases: hypoechoic or heterogeneous internal structure, irregular border, absence of hilum [10], spherical shape and, peripheral nodal blood flow pattern [11], [22]. Hypoechoic internal structure is not an unusual sonographic characteristic for cervical LN, neither is the absence of a hilum [10]. Also spherical shape is a normal finding of the LN in level 1 [22]. In the present study we successfully combined the criteria where the presence of four or five of these sonographic characteristics predicted LN metastases in seven of 51 patients. Other US predictive scoring scales have been described encompassing the following characteristics: S/L ratio, internal echo and vascular patterns [27]–[29]. However, these scoring scales require multiplication of the regression coefficient for each characteristic to obtain a total score why they are sub-optimal for general clinical implementation. Also, these patients groups had palpable enlarged cervical LNs caused by various types of cancer and benign lymphadenopathies, with the majority of the malignant LNs being more than 10 mm. The variation in aetiology and inclusion- and radiological criteria complicates the comparison of accuracy of the imaging. For example the term ‘clinical staging’ encompasses palpation, imaging or US guided FNAC. The International Union Against Cancer has proposed the use of a certainty factor (C-factor) to reflect the validity of classification according to the diagnostic method employed [30]. In the context of the present study, staging based on palpation of the neck would be categorised as C1. CT, MRI and US (and FNAC) would be categorized as C2. SNB would be categorized as C3. The pathological staging from SND would be C4 [30]. The use of the C-factor could ease comparison of studies by specifying the diagnostic staging-method employed. Also if the current imaging methods could be lifted to a higher level of validity by increasing the accuracy, the need for prophylactic treatment of the neck may be omitted. In the present study PET-CT was not included in the clinical work-up of the patients. Even though PET-CT has shown to more reliable identify metastases than CT or MRI [31], the spatial resolution of PET-CT may limit the detection of small (<5 mm) intranodal metastatic deposits [32],[33]. Currently PET-CT is not recommended as a standard diagnostic tool in staging of patients with early T stages and cN0 OSCC but is used for detection of distant metastases or second primary tumours and for irradiation planning for inoperable patients or patients with advanced stages [34]. At present none of the current imaging modalities for clinical staging; US, CT, MRI or positron emission tomography (PET)-CT seem to reliably reduce the percentage of occult metastases in patients with cN0 OSCC to less than 10% [32].

Evaluation of the inter- and intrarater reliability of the US was not possible due to logistical and ethical reasons. Therefore, evaluations of the method and of the effect of US experience in larger populations are needed. Also, future studies may validate the accuracy of FNAC in combination with the cut-off criteria proposed in the present study. Furthermore, in the present study SND and SNB are regarded equivalent with regard to pathological N-stage, which may not be absolutely correct since SNB has shown a sensitivity of 94% and NPV of 96% compared to neck dissection [35]. However, the strict inclusion criteria used may result in a high internal validity while the external validity for a general population of patients with OSCC may be limited. Newer techniques like 3D US [28], contrast enhanced US [36], [37], real-time shear wave US elastography [38], [39], and CT-perfusion [40] evolve and may prove suitable for detection of metastases in the future.

In conclusion this prospective study indicates that adding US using sonographic characteristics increases the detection of lymph node metastases. We argue that US becomes a routine examination for staging of the neck in patients classified as cN0 by CT and/or MRI in the Danish cancer pathways. In particular US appears advantageous prior to SNB by reducing the number of re-operations.
